# Nutrition label experience and consumption of transitional foods among a nationwide cohort of 42,750 Thai adults

**DOI:** 10.1108/BFJ-07-2016-0327

**Published:** 2017

**Authors:** Wimalin Rimpeekool, Martyn Kirk, Vasoontara Yiengprugsawan, Cathy Banwell, Sam-ang Seubsman, Adrian Sleigh

**Affiliations:** Research School of Population Health, Australian National University, Canberra, Australia; Research School of Population Health, Australian National University, Canberra, Australia; Centre for Research on Ageing, Health and Wellbeing, Australian National University, Canberra, Australia; Research School of Population Health, Australian National University, Canberra, Australia; School of Human Ecology, Sukhothai Thammathirat Open University, Nonthaburi, Thailand; Research School of Population Health, Australian National University, Canberra, Australia

**Keywords:** Thailand, Processed foods, Nutrition label, Nutrition transition, Socio-demographic

## Abstract

**Purpose:**

The purpose of this paper is to assess the usefulness of nutrition labels in Thailand during nutrition transition from traditional to modern diets that increase salt, sugar, and calorie intake and to note socio-demographic interactions and associations with consumption of transitional processed foods.

**Design/methodology/approach:**

The authors studied 42,750 distance learning Open University adults aged 23-96 years in 2013 residing nationwide and participating in an ongoing community-based prospective cohort study. The authors used multivariable logistic regression to relate nutrition label experiences (“read”, “good understand”, “frequent use”), socio-demographic factors, and consumption of four transitional foods. These foods included “unhealthy” instant foods, carbonated soft drinks, and sweet drinks, or “healthy” milk.

**Findings:**

Overall, two-thirds reported good understanding and frequent use of nutrition labels. Unhealthy transition-indicator processed foods were frequently consumed: instant foods (7 per cent), (carbonated) soft drinks (15 per cent), and sweet drinks (41 per cent). Frequent users of nutrition labels (e.g. females, older persons, professionals) were less likely to consume unhealthy indicator foods. Those with the most positive overall nutrition label experience (“read” + “good understanding” + “frequent use”) had the best indicator food profiles: instant foods (odds ratio (OR) 0.63; 95%CI, 0.56-0.70); soft drinks (OR 0.56; 95%CI, 0.52-0.61); sweet drinks (OR 0.79; 95%CI, 0.74-0.85); milk (OR 1.87; 95%CI, 1.74-2.00).

**Originality/value:**

Knowledge protected – those with most nutrition label experience were least likely to consume unhealthy foods. Results support government regulated nutrition labels, expanding to include sweet drinks. The study is remarkable for its large size and nationwide footprint. Study subjects were educated, represent Thais of the future, and show high awareness of transition-indicator foods.

## Introduction

Rapidly modernizing traditional societies have diets that are changing from low fat cereal-based agrarian foods to industrial processed foods, high in sodium and sugar (Kosulwat, 2002; Popkin, 1993). This “nutrition transition” creates prominent risks for increasing burdens of non-communicable diseases (NCDs) (Anderson, 2014; He and MacGregor, 2008; Karppanen and Mervaala, 2006; Lim *et al*., 2014; Popkin, 2015). Nutrient-related risks are important for diabetes, obesity, hypertension, ischaemic heart disease, and stroke. In addition, sugar and salt are often hidden ingredients in industrial processed foods that are neither sweet nor salty. Nutrition labels are promoted by governments to increase public knowledge of calorie and nutrient intakes (Codex Alimentarius Commission, 2001; Rimpeekool *et al*., 2015c). Therefore, it is important that health agencies monitor the impact of nutrition labels on food intake behaviour to provide evidence for strategies to promote healthy eating.

In Thailand, a leading South East Asian country with a middle income economy, the nutrition transition is quite advanced and NCDs are now the largest cause (71 per cent) of Thai mortality (World Health Organization, 2014). Accompanying trends show rising consumption of industrial processed foods high in sugar, calories, or sodium (Monteiro *et al*., 2010, 2011). Indeed, 20 per cent of Thai sodium consumption comes from processed foods such as instant noodles (Supornsilaphachai, 2013). Sugar sweetened beverages have been linked to longitudinal weight gain in Thailand (Lim *et al*., 2014) and are contributing to growing problems with obesity and diabetes (Popkin *et al*., 2012). Sugar consumption per person per year has tripled from 12.7 kg in 1983 to 36.6 kg in 2011 (Ministry of Public Health, 2013); sugar and salt consumption in Thailand now double the recommended intakes (Ministry of Public Health, 2011).

In other countries, the impact of nutrition labels on consumers has been related to socio-demographic factors including sex, age, and education (Campos *et al*., 2011; Drichoutis *et al*., 2006; Ranilović and Barić, 2011; Satia *et al*., 2005). Since 1998, the Thai Government has used nutrition labels as a tool to promote healthy diets among the population (Royal Thai Government Gazette, 1998). But in Thailand we know little about label effects or related socio-demographic factors associated with behavioural outcomes including geographic location, region, income, occupation, religion, and household size. Processed foods targeted for labelling are sold “prepackaged” and often “ready-to-eat”. Regulations first required nutrition information panels (NIPs) and later added guideline daily amounts (GDAs). In Thailand, NIPs and GDAs are mandated only for specific food products, rather than all. Both were created to respond to consumer concerns about nutrients in pre-packaged foods, especially sugar, fat, and sodium. NIPs and GDAs are now widespread in the Thai food market. In 2013, many “ready-to-eat” foods displayed NIPs (75 per cent) and GDAs (33 per cent) and now the percentages have increased further (Kumsri *et al*., 2013). In 2015, another government survey found that 46 per cent of sweet drinks (coffee, tea, and herbal drinks), 81 per cent of carbonated soft drinks, 66 per cent of instant foods, and 90 per cent of milk and milk products displayed nutrition labels (Pong-Utta *et al*., 2016). In 2016, instant foods were obligated to have nutrition labelling (Royal Thai Government Gazette, 2016b).

Some foods associated with the nutrition transition have become a focus of labelling because they are vectors of excess salt and sugar (Baker and Friel, 2014). For example, instant noodles are the most popular high-sodium pre-packaged food (Sinawat *et al*., 2009). Also nutritionally unhealthy are (carbonated) “soft drinks” and “sweet drinks” with added sugar (categorically separate in Thai) such as iced tea and herb drinks (Lim *et al*., 2014). In contrast, Thais view milk as healthy transitional food and promote it at school (Smitasiri and Chotiboriboon, 2003). Milk is minimally processed and at least nutritionally “neutral” and may actually protect against diabetes (Tong *et al*., 2011). The association between nutrition label experience and consumption of such transition-indicator foods – three unhealthy and one healthy – would shed light on utility of the labels but has never been investigated in Thailand.

To address this knowledge gap we studied nutrition labels and transitional foods in a large nationwide cohort that is part of our ongoing health-risk (and nutrition) transition research in Thailand. That research is focussed on emerging NCD as incomes rise, mother-child mortality falls, and nutrition transition proceeds (Sleigh *et al*., 2008). Here we report Thai nutrition label experience (reading, understanding, and using labels) and associations with the nutrition transition as represented by the four transition-indicator foods.

## Methods

This research on nutrition label experience is a sub-study within an overarching Thai cohort study (TCS) that has been described elsewhere (Seubsman *et al*., 2011, 2012; Sleigh *et al*., 2008). The TCS eight year follow-up proceeded throughout 2013 gathering repeat data on many original socio-demographic, health and behaviour variables, and including new questions about nutrition labels. Here we analyse the new data on “reading”, “understanding”, and “use” of the labels, crosslinking with other cohort data on personal socio-demographic attributes and transitional food consumption.

### Study population and data collection

The members of TCS were 87,151 home-based distance learning Sukhothai Thammathirat Open University (STOU) students residing all over Thailand. Generally cohort members displayed considerable variation of socio-economic status, lifestyle, personal behaviours, and were similar to the profile of their community. In 2005, they responded to the baseline questionnaire, representing well the Thai population for sex ratio, median age, religion, ethnicity, regional distribution, and median income (Sleigh *et al*., 2008). Also, TCS represented well the distance learning student body studying at STOU in 2005 (Seubsman *et al*., 2012). In 2005, when the Open University cohort began, the prior education level of cohort members was junior high school (4 per cent), high school (45 per cent), diploma/certificate (27 per cent), and university degree (24 per cent). In 2005, TCS members had completed more education than the general Thai population (grade 9: 100 per cent vs 43 per cent; grade 6: both 100 per cent).

Among TCS members, 60,569 (70 per cent) responded at the four year follow up in 2009 and 42,785 (71 per cent) at the eight year follow up in 2013. For each survey (baseline, four and eight year) a questionnaire was developed and pretested with small groups of on-campus STOU students. Whenever possible, standard validated questions were used. The baseline questionnaire (20-pages) collected socio-demographic, cultural, environmental, behavioural, dietary, and health information; the four and eight year questionnaires were shorter (ten pages) and made repeat observations on changeable variables and added new questions according to current research topics.

In 2013, the eight year follow-up was conducted and included new questions on nutrition labelling as well as diet indicators (see indicator foods section). We also recorded repeat data for age, sex, geographic location, urbanization, household size, education, occupation, and income. After excluding monks and prisoners (*n* = 35), who cannot go shopping, 42,750 TCS members remained for analysis.

### Study measures and definitions

#### Socio-demographic factors

In 2013, respondents fell into three age groups: 23-34, 35-49, and ≥;50 years. We noted location of residence (urban or rural), region (six categories), the number of people in the household, and income categories. Participants were studying at university in 2005 and had completed years 9-12 of high school. Occupation was elicited by the question “Which of the following best describes your primary occupation?” Most of those not responding to this question were not in paid employment or had retired. Information on religion (Buddhist, Muslim, Christian, and other/none) was obtained from the baseline survey in 2005.

#### Nutrition labels

Four questions on nutrition labels were included in the 2013 follow-up questionnaire. The first three questions focussed on key label experiences (“read”, “understand”, “use” – see below). In the fourth question we asked “Would you like to see additional nutrition labels on food products?” (yes/no).

Read “Have you ever seen nutrition labels on food products?” Responses were “seen and read”, “seen not read”, and “unaware”. Responses were dichotomized, contrasting the first experience category (“read”) with the last two experience categories (combined as “not read”).

Understand “How well do you understand the information presented on nutrition labels?” Possible responses included “understand fully”, “understand most information”, “understand some information”, “do not understand information but I know it has potential”, and “do not understand information or its potential”. The first two responses were collapsed into “good understanding” and the other three responses into “not good understanding”.

Use “How often do you use information from nutrition labels on food products to assist your food purchasing decision?” Possible responses included “every time I shop”, “often”, “sometimes”, “seldom”, and “never”. The responses were combined so that “every time” and “often” became “frequent use” and other responses as “infrequent use”.

For analysis, responses to the questions on read, understand, and use were dichotomized into coherent binary variables. This balanced cell numbers and facilitated interpretation of the results. It also enabled use of logistic regressions which were easily adjusted for covariants.

#### Indicator foods

Focussed on the nutrition transition, diet was assessed using a simplified food frequency instrument developed (in Thai) for four indicator foods – “instant foods”, “soft drinks”, “sweet drinks”, and “milk”. Examples given for instant foods were instant noodles, for soft drinks were coke and pepsi, for sweet drinks were green tea, iced coffee, and herbal drinks, and for milk were fresh, UHT, or powder milk. These four indicator foods were adapted from food items investigated in recent Thai national food consumption surveys (1995, 2003, 2009) (Aekplakorn and Steannoppakao, 2011). They also are prominent in a recent analysis of processed foods and nutrition transition in Asia (Baker and Friel, 2014). The first three indicator foods studied were considered nutritionally unhealthy because of high sodium (instant foods which are likely to be noodles) or high sugar (soft drinks or sweet drinks). The fourth indicator food was considered nutritionally healthy (milk). For each food respondents were asked: “On average how often do you consume the following types of food?” Responses scaled from “never or less than monthly”, “1-3 times/month”, “1-2 times/week”, “3-6 times/week”, and “daily or more”. For analysis, “frequent” consumption was coded for those who ate the food three or more times/week, and others were categorized as “not frequent”.

### Statistical analysis

Completed questionnaires returned by mail (*N* = 42,785) were scanned and digitized using Thai Scandevet software. Further editing used SQL and SPSS software. For analysis we used Stata v14. Individuals with missing data were excluded from analyses. We also excluded respondents from households with more than 15 people, as they may have been living in institutions (barracks, temples, prisons). We classified occupations into six groups: professional, managers, office assistants, workers, not working or retired, and unidentified occupation.

We calculated frequencies and proportions for all categorical variables (Table I) and means and standard deviations (SDs) for age (in the text). Categorical variables included socio-demographic attributes, label experience variables (read, understand, and use), and indicator food intakes (instant foods, soft drink, sweet drink, milk).

We constructed multivariable logistic regression models showing the independent effects of the mutually adjusted socio-demographic variables. The dependent variables were the label experiences (three outcomes – Table II) and the indicator food intakes (four outcomes – Table III). Correlation coefficients among independent variables were calculated and were less than 0.6. For each of the seven models, odds ratios (ORs) and 95 per cent confidence intervals were estimated for the socio-demographic factors.

Finally, we estimated associations between label experience variables and consumption of the four indicator foods (four models – Table IV). To do this, we used the three label experiences (read, understanding, use) to produce a combined Code (1-5) as follows: (1) “not read” (regardless of understanding or use); (2) read, “not good” understanding, and “infrequent” use; (3) read, “good” understanding but “infrequent” use; (4) read, “not good” understanding, and “frequent” use; (5) read, “good” understanding, and “frequent” use. Then for each indicator food outcome we modelled the independent effect of the code and adjusted for all socio-demographic factors. All multivariable models were saturated (i.e. included all variables assessed) because we found that the ORs and 95 per cent confidence intervals did not change much when non-significant variables were removed. This stability of our effect estimates is a result of the large sample size. Our final models contained all the potential explanatory variables with OR estimates mutually adjusted for the statistical influence of all other variables in the model.

### Ethical approval

Ethics approval was obtained from Sukothai Thammathirat Open University Research and Development Institute (protocol 0522/10) and the Australian National University Human research Ethics Committee (protocols 2004/344 and 2009/570). Informed written consent was obtained from all participants.

## Results

Overall, responses of 42,750 cohort members were analysed for the eight year survey, including 19,295 men (45.1 per cent) and 23,455 women (54.9 per cent). The mean ± SD age was 40.5 ± 8.5 years, 6.0 per cent lived alone, 55.3 per cent lived in an urban environment, and the most frequent household size was 2-4 persons. Participants resided all over Thailand with the largest groups located in the central-east (30.7 per cent) or Bangkok (15.8 per cent). Most of the cohort (79.8 per cent) was university educated and the most frequent occupations were “professional” (26.4 per cent), or “office assistant” (30.8 per cent). Monthly incomes were modest, with nearly 60 per cent reporting 20,000 baht (approximately USD$550) or less per month. Responses to the nutrition label questions indicated 89.0 per cent had “read”, 69.5 per cent had a “good understanding”, and 64.4 per cent had “frequent use”. Almost everyone (96.4 per cent) “wanted to see additional nutrition labels”. The participants also reported frequent consumption of indicator foods – instant foods (7.0 per cent), soft drinks (14.6 per cent), other sweet drinks (40.7 per cent), and milk (45.5 per cent) (Table I).

Socio-demographic characteristics were examined for bivariate associations with nutrition label outcomes (read, good understanding, and frequent use). Overall, age, sex, location, region, religion, household size, education, occupation, and income were all significantly associated (*p* < 0.05) with at least one label outcome. When explored further, associations for age, sex, location, region, and occupation were found to be strongly connected (*p* < 0.001) to at least two of the outcomes.

In multivariable analyses of the three dependent label experience variables (Table II), adjusted for covariates, female participants had “read” labels more (OR 1.79; 95% CI, 1.68-1.92), and “used” them more frequently (OR 1.65; 95% CI, 1.58-1.73). Increasing age associated with reading, good understanding, and frequent use of labels with ORs ranging from 1.17 to 1.57. Living in an urban location was associated with less label “reading” (OR 0.86; 95% CI, 0.81-0.93) and less “good understanding” (OR 0.89; 95% CI, 0.85-0.93) but had no association with “frequent use” of labels. Compared to participants in central-east Thailand, Bangkok residents “read” labels less, had less “good understanding” and reported less “frequent use” with ORs ranging from 0.88 to 0.92. In contrast, people in Southern Thailand reported they “read” labels more, had a “good understanding”, and had more “frequent use” with ORs ranging from 1.20 to 1.25. Thai Muslims also “read”, “understood”, and “frequently used” nutrition labels a little more than the Buddhist group but only the greater use of labels was significant. Some occupations associated with label outcomes, especially professionals, whose adjusted ORs for the three label outcomes ranged from 1.10 to 1.30. Monthly income had little association with label outcomes after adjusting for all other covariates.

Multivariable analysis of independent socio-demographic factors and the four dependent indicator food outcomes (Table III) showed female participants had less frequent consumption of instant foods (OR 0.68; 95% CI, 0.63-0.74), soft drinks (OR 0.62; 95% CI, 0.59-0.66), and sweet drinks (OR 0.79; 95% CI, 0.76-0.83), but more frequent consumption of milk (OR 1.67; 95% CI, 1.60-1.74). Increasing age and rural residence associated with less frequent consumption of all indicator foods, as did residence in the southern region. University educated participants were significantly less likely to consume instant foods and soft drinks, but not sweet drinks and milk. There was a strong inverse association between income and frequent consumption of instant foods.

Finally, we analysed the associations of overall label experience, combining the three experience variables into one composite code (Table IV). People who only read nutrition labels (without good understanding or frequent use) were significantly less likely to frequently consume instant foods and soft drinks, but not sweet drinks, and were significantly more likely to frequently drink milk. Beyond reading labels, “frequent use” was associated with lower ORs of frequent consumption for instant foods, soft drinks, and sweet drinks (ORs range from 0.56 to 0.87) and higher OR for milk intake (OR 1.63; 95% CI, 1.49-1.78). Respondents with the most label experience – “reading” plus “good understanding” plus “frequent use” – had the strongest association with indicator foods, lowering ORs for frequent instant foods (OR 0.63; 95% CI, 0.56-0.70), soft drinks (OR 0.56; 95% CI, 0.52-0.61), and sweet drinks (OR 0.79; 95% CI, 0.74-0.85) while boosting the OR for frequent consumption of milk (OR 1.87; 95% CI, 1.74-2.00).

## Discussion

This Thai study systematically assesses the value of nutrition label experience and its association with food consumption. The results enlighten an under-researched area – nutrition label use and changing diets in South East Asia. The topic is important and Thailand is a regional leader in the ongoing nutrition transition. These countries share similar food cultures and some are contemplating the introduction of nutrition labels to combat the transition’s health effects.

Except for their generally higher education, the 42,750 cohort adults who participated in our study were geographically and socio-demographically similar to the general Thai population. Overall, 89 per cent of the cohort reported “reading” nutrition labels and about two-thirds reported “good understanding” or “frequent use”, so for all three experiences nutrition labels were reaching the study population. Females, those age 50 years or more, and rural or southern residents were the socio-demographic groups with strongest positive statistical associations with nutrition label experience (read, understand, use). As well, these groups had less frequent consumption of unhealthy indicator foods (instant foods, carbonated soft drinks, and sweet drinks) and more frequent consumption of (healthy) milk. These relationships persisted after adjusting for many covariates.

Our findings agree with international studies that show women tend to have better diets than men and are more likely to eat fruit and fibre, avoid high-fat foods, and limit salt (Wardle *et al*., 2004) and are more likely to read and use nutrition labels (Campos *et al*., 2011). This gender differential is attributed to negative social and psychological effects from obesity (Ferguson *et al*., 2009) and also to greater interest in health. We also found that older adults were more likely to use nutrition labels than others, a result that contrasted with the majority of studies (Campos *et al*., 2011). However, older Americans use labels significantly more (*p* < 0.01) than younger persons (Stran and Knol, 2013). Chronic diseases usually appear with ageing and may spark an increased interest in healthy diets and label use (Andreas and Panagiotis, 2005).

Our study also found that Thai cultural geography interacts with nutrition labelling. Bangkok respondents were substantially less likely to read them compared to respondents from all other regions. We also found little difference in the nutrition label use for rural and urban Thais in sharp contrast to a US report showing 40 per cent less use for rural adults (Chen *et al*., 2012). Indeed, rural Thais may have better nutrition behaviour than urban counterparts as urbanization leads to dietary transition to processed foods (Kelly *et al*., 2010). In Thailand, rural people are less overweight than urban people (Aekplakorn *et al*., 2007). Recent nationwide research using a random sub-sample of the TCS showed that 85 per cent do some shopping in supermarkets that sell pre-packaged processed foods high in salt, fats, and sugars. However, Thai rural residents retain good access to fresh food markets although supermarkets selling labelled packaged goods, are expanding rapidly in these areas and fresh food markets are receding in cities (Kelly *et al*., 2014). This transition points to an urgent need for nutrition labelling to help Thais understand the content and healthiness of their newly adopted diets.

We also observed regional differences with the highest odds for reading nutrition labels in the Southern region and in the North. Notably these two culturally distinctive regions also had the highest fruit and vegetable consumption in Thailand reported by the National Health Examination Survey IV in 2009 (National Health Examination Survey Office, 2009). As well, we noted a tendency for Muslims to use nutrition labels a little more than others. This could reflect compliance with Islamic dietary restrictions. So in Thailand both culture and religion are associated with nutrition label use.

We found that education level had a positive statistical association with label experience and higher education associated with less frequent consumption of instant foods and soft drinks. But we did not have much variation of education due to the nature of our cohort. However, in another (qualitative) study of nutrition label use among Thai consumers, we found other label attributes could mediate education effects including readability, technical jargon, unobtrusive location, and suspected truthfulness (Rimpeekool *et al*., 2015b). We also found education must align with positive attitudes and accepting beliefs to motivate use (Rimpeekool *et al*., 2015a). As well trust in the safety and quality of the food supply could influence Thai consumers who feel more confident of traditional (unlabelled) food from fresh markets (Banwell *et al*., 2016). A recent systematic review of trust in food supply systems shows research on this important topic remains very limited (Tonkin *et al*., 2015).

We found professional people and managers were more likely than others to understand and use nutrition label information and were less likely to report frequent consumption of instant foods. A recent report from Canada showed low socio-economic status associated with poor label comprehension (Sinclair *et al*., 2013). High income earners reported lower consumption of instant foods and soft drinks. Others have reported that higher income associates with increased vegetable or fruit intakes as these products are purchased for their healthiness rather than value for money (Konttinen *et al*., 2013; Satheannoppakao *et al*., 2009).

This report complements a recent National Food Consumption Survey of Thailand in 2009 which produced similar consumption frequencies for instant foods, soft drinks, and sweet drinks (after allowing for methodological differences) (Aekplakorn and Steannoppakao, 2011). Our report also supports two earlier unpublished surveys each based on random samples of 2,000 people drawn from all regions, with estimates for label understanding for both NIP and GDA of about 60 per cent (Food and Drug Administration Thailand, 2010; Yodtheun *et al*., 2013).

Some limitations and strengths of our study should be noted. First, participants were educated so for outcomes related to education level it was not possible to generalize results. Otherwise, cohort members were socio-demographically similar to the Thai population. Second, data are based on self-administered responses to mailed questionnaires but cohort members are used to complex information received by mail. Questionnaires were quite long (10-20-pages) so special interest in one or two questions would have little influence on overall responses (Chen *et al*., 2012). Generally we have found that study drop out from TCS is related to residential mobility and not to health outcomes (Sleigh *et al*., 2008). Third, our qualitative study, based on in-depth 30-45 minute interviews, produced supportive information (Rimpeekool *et al*., 2015a). As well, further support comes from formal validations of several TCS questionnaire responses including weight, height, waist circumference, medical outcome Short Form 36, and hypertension (Lim *et al*., 2008, 2009, 2012; Thawornchaisit *et al*., 2014). Fourth, we do not have direct information on food purchases. However, other studies have found that nutritional label use contributes to healthier food consumption or reduced consumption of “unhealthy” foods (Azman and Sahak, 2014; Drichoutis *et al*., 2006; Guthrie *et al*., 1995; Kreuter *et al*., 1997; Wills *et al*., 2009).

The nutrition transition risks considered in this study relate to high intake of sugar and sodium, especially noted among males, urban dwellers, the less educated, and those with lower monthly income. These groups interact less with nutrition labels and have less healthy diets. Nutrition label education and health promotion should target these groups to increase understanding and stimulate healthy eating behaviour. Also, sweet drinks should now be required to have nutrition labels. Our previous qualitative research shows that Thai nutrition labels can be improved for readability and understanding in line with the improved labels launched recently by the USA (US Food and Drug Administration, 2016). We also note that other nutrition interventions are coming to Thailand. MOPH now has a “Health Logo” which approved foods can display (Royal Thai Government Gazette, 2016a) and the Thai Food and Drug Administration proposes a sugar tax (Sattaburuth, 2016).

Further studies could help nutrition labelling policies for Thai consumers. These include the revision of nutrient and serving size reference values and investigation of Thai consumers for visual attention and cognitive processes in relation to labels, testing new research methods such as “eye-tracking technology”. Overall, we need a deeper understanding of label experiences in relation to health knowledge, motivation, and psychology. We will then be in a position to explain and modify food-related behaviour. As well we need a better understanding of the industrial impact of nutrition labelling regulations and that will require systematic study of all the main categories of processed food manufacturers.

## Conclusion

Our nationwide study of nutrition labels in transitional Thailand showed most respondents read the labels but fewer used the information. Our study participants were of modest means but were well educated. Socio-demographic factors (e.g. income, sex) strongly associated with nutrition label experiences (read, understand, use) and frequent intake of indicator foods typical of the nutrition transition (instant foods, soft and sweet drinks, milk). Nutrition label experiences were strongly and significantly associated with consumption of transition-indicator foods. These results arise in a South East Asian country that recently defeated malnutrition but now confronts an equally important new community nutrition challenge (Chavasit *et al*., 2013; Kosulwat, 2002). Overall, our study supports the use of nutrition labels in Thailand and lends weight to the government’s planned introduction of mandatory NIP on all pre-packaged foods.

## Figures and Tables

**Table I:** Socio-demographic attributes, nutrition label outcomes and indicator food intakes of Thai cohort in 2013

Attributes	*n* a	%^b^	Attributes	*n* a	%^b^
*Sex*			*Household size (people)*		
Male	19,295	45.1	1	2,513	6.0
Female	23,455	54.9	2-4	26,306	62.4
*Age group (years)*			5-15	13,350	31.7
23-34	12,127	28.4	*Education*		
35-49	23,984	56.1	Non university	8,603	20.2
≥50	6,639	15.5	University	33,925	79.8
*Location*			*Occupation*		
Rural	18,913	44.7	Worker	8,044	18.9
Urban	23,434	55.3	Manager	6,023	14.2
*Region*			Professional	11,228	26.4
Central-East	13,107	30.7	Office assistant	13,068	30.8
Bangkok	6,741	15.8	Not working/retired	2,757	6.49
North	8,580	20.1	Unidentified	1,370	3.22
Northeast	8,954	21.0	*Monthly income (baht)*		
South	5,368	12.6	<10,000	9,378	22.2
*Religion* c			10,001-20,000	15,831	37.4
Buddhist	40,293	94.6	20,001-30,000	9,234	21.8
Muslim	1,491	3.5	>30,000	7,853	18.6
Christian	746	1.8			
Other/none	72	0.2			
*Nutrition label outcomes*				*n* a	%^b^
Nutrition labels on food?				37,914	89.0
Read				4,708	11.1
Not read					
Understand the information on “nutrition labels”				29,452	69.5
Good				12,917	30.5
Not good					
Use nutrition labels to assist food purchasing?					
Frequent use				27,457	64.4
Infrequent use				15,173	35.6
Like to see additional nutrition labelling on foods?					
Yes				40,296	96.4
No				418	1.0
Not sure				1,076	2.6
*Frequent consumption of indicator foods (≥3 times/week)*				*n* a	%^b^
Instant foods				2,966	7.0
Soft drinks				6,169	14.6
Sweet drinks				17,277	40.7
Milk				19,307	45.5

**Notes:** *n*=42,750.

a Sample size may not add to 42,750 due to missing data (0.3-1.1 per cent of variables had missing values);

b some percentages may not equal 100 due to rounding;

c information on religion obtained from the 2005 TCS baseline survey

**Table II:** Multivariable logistic regression associating socio-demographic characteristics with nutrition label experience

	Nutrition label experience (OR, 95%CI)		
Socio-demographic characteristics	Read	Good understanding	Frequent use
*Sex*			
Male	1.0	1.0	1.0
Female	1.79 (1.68-1.92)***	1.01 (0.97-1.06)	1.65 (1.58-1.73)***
*Age group (years)*			
23-34	1.0	1.0	1.0
35-49	1.19 (1.11-1.28)***	1.17 (1.12-1.23)***	1.22 (1.16-1.28)***
≥50	1.19 (1.07-1.32)**	1.57 (1.45-1.69)***	1.39 (1.29-1.49)***
*Location*			
Rural	1.0	1.0	1.0
Urban	0.86 (0.81-0.93)***	0.89 (0.85-0.93)***	0.97 (0.93-1.02)
*Region*			
Central-East	1.0	1.0	1.0
Bangkok	0.91 (0.83-1.00)*	0.88 (0.82-0.94)***	0.92 (0.86-0.98)*
North	1.20 (1.10-1.32)***	1.20 (1.12-1.27)***	1.31 (1.23-1.39)***
Northeast	1.14 (1.04-1.25)**	1.12 (1.05-1.19)***	1.24 (1.17-1.32)***
South	1.25 (1.11-1.40)***	1.20 (1.11-1.29)***	1.21 (1.13-1.31)***
*Religion*			
Buddhist	1.0	1.0	1.0
Muslim	1.09 (0.90-1.32)	1.04 (0.92-1.18)	1.15 (1.02-1.30)*
Christian	0.84 (0.67-1.05)	1.09 (0.92-1.28)	0.98 (0.83-1.14)
Other/no religion	1.12 (0.53-2.35)	1.26 (0.74-2.14)	0.74 (0.46-1.20)
*Household size (people)*			
1	1.0	1.0	1.0
2-4	0.95 (0.84-1.09)	1.02 (0.93-1.12)	0.94 (0.86-1.03)
5-15	0.97 (0.84-1.11)	1.01 (0.91-1.11)	1.00 (0.91-1.09)
*Education*			
Non university	1.0	1.0	1.0
University	1.05 (0.97-1.14)	1.14 (1.08-1.21)***	0.96 (0.91-1.02)
*Occupation*			
Worker	1.0	1.0	1.0
Manager	0.98 (0.87-1.09)	1.17 (1.08-1.26)***	1.08 (1.00-1.17)*
Professional	1.10 (1.00-1.23)	1.30 (1.21-1.40)***	1.17 (1.09-1.25)***
Office assistant	0.92 (0.84-1.01)	0.93 (0.88-0.99)*	0.99 (0.93-1.05)
Not working/retired	1.16 (0.99-1.35)	1.08 (0.97-1.19)	1.08 (0.98-1.19)
Unidentified	1.09 (0.89-1.32)	1.16 (1.02-1.33)*	1.26 (1.11-1.43)***
*Monthly income (baht)*			
<10,000	1.0	1.0	1.0
10,001-20,000	1.01 (0.92-1.10)	0.98 (0.92-1.04)	0.98 (0.92-1.04)
20,001-30,000	1.02 (0.92-1.13)	1.07 (1.00-1.15)	1.00 (0.93-1.07)
>30,000	1.05 (0.94-1.18)	1.17 (1.08-1.27)***	1.01 (0.93-1.09)

**Notes:** *n*=42,750. Models are adjusted for all socio-demographic characteristic.

* *p*<0.05;

** *p*<0.01;

*** *p*<0.001

**Table III:** Multivariable association (OR, 95%CI) of socio-demographic characteristics with frequent consumption of indicator foods

	Frequent consumption (≥3 times/week)			
Socio-demographic characteristics	Instant food	Soft drink	Sweet drink	Milk
*Sex*				
Male	1.0	1.0	1.0	1.0
Female	0.68 (0.63-0.74)***	0.62 (0.59-0.66)***	0.79 (0.76-0.83)***	1.67 (1.60-1.74)***
*Age group (years)*				
23-34	1.0	1.0	1.0	1.0
35-49	0.63 (0.58-0.68)***	0.55 (0.51-0.58)***	0.83 (0.79-0.87)***	0.75 (0.72-0.79)***
≥50	0.29 (0.24-0.34)***	0.28 (0.25-0.31)***	0.52 (0.49-0.56)***	0.72 (0.67-0.77)***
*Location*				
Rural	1.0	1.0	1.0	1.0
Urban	1.11 (1.02-1.21)*	1.27 (1.20-1.36)***	1.19 (1.13-1.24)***	1.00 (0.96-1.05)
*Region*				
Central-East	1.0	1.0	1.0	1.0
Bangkok	0.98 (0.86-1.11)	1.00 (0.92-1.09)	1.15 (1.08-1.22)***	0.99 (0.93-1.06)
North	1.17 (1.05-1.30)**	0.45 (0.41-0.49)***	0.82 (0.77-0.87)***	1.09 (1.03-1.15)**
Northeast	1.21 (1.09-1.35)***	0.82 (0.76-0.89)***	0.87 (0.82-0.92)***	1.05 (0.99-1.11)
South	0.77 (0.66-0.90)**	0.30 (0.26-0.34)***	0.79 (0.73-0.85)***	0.94 (0.88-1.01)
*Religion*				
Buddhist	1.0	1.0	1.0	1.0
Muslim	1.33 (1.07-1.64)**	0.82 (0.67-1.00)	1.07 (0.95-1.20)	1.17 (1.04-1.32)**
Christian	1.26 (0.97-1.66)	1.03 (0.83-1.28)	0.92 (0.79-1.08)	0.85 (0.73-0.99)*
Other/no religion	2.79 (1.50-5.20)***	2.23 (1.31-3.80)***	1.35 (0.84-2.19)	1.07 (0.66-1.73)
*Household size (people)*				
1	1.0	1.0	1.0	1.0
2-4	0.76 (0.66-0.89)**	1.06 (0.94-1.20)	0.92 (0.85-1.00)	0.94 (0.87-1.03)
5-15	0.81 (0.69-0.95)**	1.21 (1.07-1.38)**	0.97 (0.89-1.06)	0.91 (0.83-1.00)*
*Education*				
Non university	1.0	1.0	1.0	1.0
University	0.78 (0.71-0.85)***	0.83 (0.77-0.89)***	0.95 (0.90-1.00)	0.99 (0.94-1.05)
*Occupation*				
Worker	1.0	1.0	1.0	1.0
Manager	0.86 (0.74-1.00)*	1.12 (1.01-1.25)*	1.00 (0.93-1.08)	0.93 (0.87-1.01)
Professional	0.87 (0.77-0.99)*	0.95 (0.86-1.04)	0.90 (0.84-0.96)**	0.91 (0.85-0.97)**
Office assistant	0.94 (0.84-1.05)	1.01 (0.93-1.10)	0.93 (0.88-0.99)*	0.86 (0.81-0.92)***
Not working/retired	0.85 (0.71-1.01)	0.90 (0.78-1.03)	0.77 (0.70-0.85)***	1.05 (0.95-1.15)
Unidentified	0.77 (0.60-0.99)*	0.97 (0.82-1.16)	0.86 (0.76-0.97)*	0.92 (0.81-1.04)
Monthly income (baht)				
<10,000	1.0	1.0	1.0	1.0
10,001-20,000	0.88 (0.79-0.97)*	0.99 (0.92-1.08)	1.09 (1.02-1.15)**	1.04 (0.98-1.10)
20,001-30,000	0.64 (0.56-0.73)***	0.88 (0.80-0.97)*	1.08 (1.01-1.16)*	0.96 (0.90-1.03)
>30,000	0.44 (0.37-0.52)***	0.82 (0.74-0.92)**	0.99 (0.92-1.07)	0.98 (0.91-1.06)

**Notes**: *n*=42,750. Models are adjusted for all socio-demographic characteristics

* *p*<0.05,

** *p*<0.01,

*** *p*<0.001

**Table IV:** Multivariable associations of combined label experience with indicator food intake^a^

Label experience			Combined Code	Odds ratio for frequent consumption of indicator food (≥3 times/week)^b^			
Read	Understand	Use		Instant food	Soft drink	Sweet drink	Milk
0	n/a	n/a	(1)	1.0	1.0	1.0	1.0
1	0	0	(2)	0.75 (0.65-0.87)***	0.79 (0.71-0.88)***	0.98 (0.91-1.07)	1.19 (1.10-1.30)***
1	1	0	(3)	0.75 (0.65-0.87)***	0.83 (0.75-0.92)***	0.95 (0.88-1.03)	1.31 (1.21-1.43)***
1	0	1	(4)	0.71 (0.61-0.83)***	0.56 (0.50-0.63)***	0.87 (0.80-0.95)**	1.63 (1.49-1.78)***
1	1	1	(5)	0.63 (0.56-0.70)***	0.56 (0.52-0.61)***	0.79 (0.74-0.85)***	1.87 (1.74-2.00)***

**Notes**:

a The label experience for each descriptive variable (read, understand, use) is shown in binary form (0=no, 1=yes). The code reveals the combines label experience as follows: if “read” = 0, Code = (1) (“understand” or “use” are then not applicable or n/a); if “read” = 1, code for each possible combination =(2)-(5);

b the model for each indicator food outcome is adjusted for all socio-demographic characteristics.

* *p* < 0.05;

** *p* < 0.01;

*** *p* < 0.001
